# Combining procalcitonin, c-reactive protein, and white blood cell count in predicting infections in pediatric open cardiac surgery with cardiopulmonary bypass

**DOI:** 10.1016/j.jped.2025.04.006

**Published:** 2025-05-17

**Authors:** Tuan Manh Ha, Man Minh Tran, Tung Viet Le, Nguyen The Nguyen Phung

**Affiliations:** a University of Medicine and Pharmacy at Ho Chi Minh City, Ho Chi Minh City, Vietnam; b University Medical Center Ho Chi Minh City, Ho Chi Minh City, Vietnam; c City Children’s Hospital, Ho Chi Minh City, Vietnam

**Keywords:** Procalcitonin, Postoperative infections, Pediatric, Open-heart surgery, Cardiopulmonary bypass

## Abstract

**Objective:**

This study aimed to evaluate the validity of models using Procalcitonin (PCT) alone and PCT combined with other biomarkers to predict early infection after pediatric open-heart surgery with cardiopulmonary bypass (CPB).

**Methods:**

A prospective observational study was conducted on children undergoing open-heart surgery with CPB, without preoperative infection. Procalcitonin, C-reactive protein (CRP), and white blood cell (WBC) count were measured preoperatively and on postoperative days 1 and 3. Postoperative infection was defined according to the Centers for Disease Control and Prevention 2008 criteria.

**Results:**

Fifty eligible cases were included, comprising 46 % males with a median age of 7 months (4–17). The AUC (area under the curve) for PCT on postoperative day 3 was 0.67 (0.51–0.82) (*p* = 0.085). The AUCs for the models combining PCT + CRP and PCT + WBC were 0.71 (0.57–0.86) (*p* = 0.014) and 0.72 (0.55–0.86) (*p* = 0.014), respectively. The AUC for the model combining PCT + CRP + WBC was 0.81 (0.69–0.93) (*p* = 0.002). The combination of PCT > 4.15 ng/ml, CRP > 22.03 mg/l, and WBC > 15.3 × 10^3^/µl predicted infection with a hazard ratio 9.66 times (2.94–31.72) higher than PCT > 4.15 ng/ml alone (*p* < 0.05).

**Conclusions:**

PCT measurement on the third postoperative day alone cannot predict infection in pediatric open-heart surgery with CPB. The combination of PCT with CRP and WBC may enhance early infection prediction, although further validation in larger, multicenter cohorts is warranted.

## Introduction

Postoperative infection in pediatric patients undergoing cardiac surgery with cardiopulmonary bypass (CPB) is one of the most common and severe complications, despite advances in surgical techniques and infection control measures.^1^^,^^2^ Postoperative infection prolongs hospital stays, increases treatment costs, and can lead to higher mortality rates.^3^ Therefore, early diagnosis of postoperative infection plays a crucial role in improving the prognosis of congenital heart disease requiring surgical intervention. However, pediatric cardiac surgery with CPB often triggers an inflammatory response, which is more severe than that in adults.^4^^,^^5^ The systemic inflammatory response in children presents clinical symptoms similar to infection.^6^ This phenomenon renders early diagnosis of postoperative infection in pediatric patients undergoing open-heart surgery with CPB particularly challenging. Therefore, reliable biomarkers are needed to differentiate between inflammation and infection in pediatric patients undergoing open-heart surgery with CPB.

Procalcitonin (PCT), the precursor of the calcitonin hormone, is a 116-amino acid protein encoded by the CALC-1 gene. Under normal physiological conditions, PCT is produced by C-cell thyroid cells and rapidly converted to calcitonin, so its circulating levels in the blood are usually undetectable. During infection and systemic inflammatory response, PCT is produced by non-thyroid cells and is not converted to calcitonin, resulting in increased blood PCT levels.^7^ Procalcitonin has been demonstrated to be a valuable biomarker for distinguishing between infection and systemic inflammatory response.^7^, ^8^, ^9^ Several studies have shown PCT to be a promising biomarker for diagnosing postoperative infection in pediatric patients undergoing open-heart surgery with CPB.^10^, ^11^, ^12^, ^13^, ^14^, ^15^, ^16^ However, some studies have reported indefinite results regarding the diagnostic role of PCT tests in postoperative infection in pediatric patients undergoing open cardiac surgery with CPB.^14^^,^^17^^,^^18^ This may result from the intense inflammatory response following CPB in children, which can cause PCT levels to increase in cases of systemic inflammatory response despite infection not occurring.^17^^,^^19^^,^^20^ Some studies suggest the need to combine PCT with other biomarkers or clinical signs to enhance the accuracy of infection diagnosis.^11^^,^^21^, ^22^, ^23^, ^24^ The American Association for Clinical Chemistry also recommends against using PCT as a stand-alone test for diagnosing infection in children.^7^ However, findings from studies on combined biomarker models for diagnosing infection remain inconsistent, and their clinical applicability is limited. This study aims to evaluate the validity of a combined model involving PCT levels, C-reactive protein (CRP) levels, and white blood cell (WBC) for predicting early postoperative infection in pediatric patients undergoing open-heart surgery with CPB.

## Materials and methods

### Study design and setting

A prospective observational study was conducted from January 2022 to December 2022 at the Pediatric Intensive Care Unit of City Children’s Hospital. A post hoc power analysis indicated that the sample size of 50 was sufficient to detect a moderate effect size with a power of 80 % at alpha = 0.05.

### Study participants

Patients were included in the study based on the following criteria: 1) age <16 years; 2) scheduled for open-heart surgery with CPB; 3) had no evidence of infection preoperatively; 4) informed consent obtained from parents or guardians. Exclusion criteria included 1) patients currently using corticosteroids or immunosuppressive drugs; 2) severe concurrent illnesses or conditions affecting patient health during sample collection for the study.

### CPB procedure

All patients underwent general anesthesia via endotracheal intubation following hospital guidelines. Prior to CPB initiation, patients were administered a priming solution containing sodium bicarbonate, mannitol, albumin, heparin (300 IU/kg), and Solu-Medrol (30 mg/kg). Red blood cells were transfused as necessary to maintain hematocrit levels between 32 % and 34 %. Cefazolin (15 mg/kg) was administered 30 min preoperatively, repeated if surgery exceeded 2 h, and then every 8 hours for 48 hours postoperatively. A cardiopulmonary bypass was performed using a Stockert S3 heart-lung machine. The oxygenator was the Capiox RX (Terumo, Japan), sized according to patient age and weight. Myocardial protection was achieved with Custodiol cardioplegia solution and mild hypothermia (28–32 °C). The flow rate was maintained at 2.2–2.4 l/min/m^2^. Ultrafiltration was routinely used during CPB to remove excess fluid and maintain fluid balance. After completing the heart surgery, patients were gradually weaned off CPB according to the cardiac surgery unit protocols. All patients were then transferred to the PICU for postoperative care and monitoring.

### Measurement of biomarkers

Procalcitonin levels were measured using the immunoenzymatic method with Access PCT reagent (Beckman Coulter, France) on a DxI 800 analyzer (Beckman Coulter, USA). The lowest detection threshold was ≤0.01 ng/ml. CRP levels were measured using the immunoturbidimetric method with CRP Latex reagent (Beckman Coulter, Ireland) on a DxC 700 AU analyzer (Beckman Coulter, Japan). The lowest detection threshold was 0.05 mg/l. WBC counts were determined using an automated analyzer, XN-2000 (Sysmex, Japan). Biomarker measurements were obtained on the preoperative day, postoperative day (POD) 1, and POD 3.

### Definition of postoperative infection

Postoperative infections were identified using CDC (Centers for Disease Control and Prevention) 2008 criteria^25^ and confirmed through clinical signs and microbiological testing (e.g., blood, sputum, and urine cultures), wherever applicable. Infection adjudication was performed independently by two physicians blinded to biomarker results, and any disagreement was resolved by a senior physician.

### Data collection

Patient-related information was recorded on standardized data collection forms. Variables included preoperative factors such as age, gender, weight, Risk Adjustment for Congenital Heart Surgery (RACHS-1) score, and preoperative hospital stay duration; intraoperative variables such as surgical approach, surgery duration, CPB time, and aortic cross-clamp time; intraoperative diagnosis; and postoperative variables such as the duration of mechanical ventilation and PICU stay. PCT, CRP, and WBC count were measured at three-time points: on the preoperative day, POD 1, and POD 3.

### Ethical considerations

This study was approved by the Ethics Committee of Hospital No. 29/QD-BVNDTP on January 17, 2022. All participants had their parents or guardians sign informed consent forms. Parents or guardians were provided with comprehensive information about the study, their rights, and potential risks. They had the right to withdraw their child from the study at any time without consequences. Participant-related information was encoded and kept confidential.

### Statistical analysis

Categorical variables are presented as frequencies and percentages. Continuous variables are presented as mean ± standard deviation (SD) for normally distributed data or as the median and interquartile range (IQR) for non-normally distributed data. Between-group comparisons of variables were performed using the Chi-square test, Fisher's exact test, or Mann-Whitney U test, where appropriate. Three biomarker models on POD 3 were used to predict postoperative infections. The cut-off values of the biomarkers in each model were obtained from the ROC curve analysis of this study. To assess the validity, receiver operating characteristic (ROC) curve analysis was used to determine the area under the curve (AUC), cut-off points, sensitivity, specificity, negative predictive value (NPV), and positive predictive value (PPV). Kaplan-Meier analysis and hazard ratio determination were used to assess and compare the validity of each model in predicting infections. Given the limited number of biomarkers, adjustment for multiple comparisons was not performed. A two-tailed p-value < 0.05 was considered statistically significant. Statistical analyses were performed using STATA version 17.

## Results

### Characteristics of the study population

Table 1 shows the characteristics of the cohort. Fifty eligible cases were recruited. Thirty-three cases (66 %) of the cohort were identified with postoperative infections according to CDC 2008 criteria. These included 25 cases of pneumonia, 4 cases of bloodstream infection, 2 cases of surgical site infection, and 2 cases of urinary tract infection. Among them, positive culture results were obtained in 8 sputum samples, 2 blood samples, and 1 urine sample. No statistically significant differences were observed in preoperative, intraoperative, or postoperative patient characteristics between the groups with and without infection (*p* > 0.05) (Table 1).

**Table 1:** Baseline characteristics of the study population.

Variables	Overall (*n* = 50) (*n*, %)	Postoperative Infection		*P*-value
		Yes (*n* = 33) (*n*, %)	No (*n* = 17) (*n*, %)	
Sex				
Male *(n, %)*	23 (46.0)	18 (54.5)	5 (29.4)	0.091^a^
Female *(n, %)*	27 (54.0)	15 (45.5)	12 (70.6)	
Age (*months*)	7	6	9	0.428^c^
*median (IQR)*	(4–17)	(4–14)	(5–17)	
Weight *(kg)*	6.45	6.3	6.7	0.789^c^
	(5.2–9)	(5.3–9)	(5.2–9.4)	
Malnutrition *(Yes)*	25 (50.0)	16 (48.5)	9 (52.9)	0.765^a^
RACHS-1				
1	3 (6.0)	1 (3.0)	2 (11.8)	0.520^b^
2	46 (92.0)	31 (94.0)	15 (88.2)	
4	1 (2.0)	1 (3.0)	0 (0.0)	
Length of preoperative hospital stay *median (IQR) (days)*	5 (3–7)	5 (3–7)	6 (4–7)	0.541^c^
Etiology				
Atrial septal defect *(n,%)*	3 (6.0)	1 (3.0)	2 (11.8)	0.264^b^
Tetralogy of Fallot *(n,%)*	16 (32.0)	12 (36.4)	4 (23.5)	0.357^a^
Ventricular septal defect *(n,%)*	24 (48.0)	17 (51.5)	7 (41.2)	0.488^a^
Single ventricle *(n,%)*	1 (2.0)	1 (3.0)	0 (0.0)	1.000^b^
TAPVR *(n,%)*	2 (4.0)	1 (3.0)	1 (5.9)	1.000^b^
Ebstein anomaly *(n,%)*	2 (4.0)	1 (3.0)	1 (5.9)	1.000^b^
Atrioventricular septal defect *(n,%)*	2 (4.0)	1 (3.0)	1 (5.9)	1.000^b^
Intraoperative period				
Surgical incision				
Lateral thoracotomy *(n, %)*	11 (22.0)	10 (30.3)	1 (5.9)	0.073^b^
Median sternotomy *(n, %)*	39 (78.0)	23 (69.7)	16 (94.1)	
Operating time *(minutes)* *median (IQR)*	230 (190–270)	230 (187.5–275)	220 (190–260)	0.760^c^
CPB time (*minutes*) *median (IQR)*	135 (110–170)	140 (114–194)	123 (101–163)	0.110^c^
Aortic cross-clamp time (*minutes*) *median (IQR)*	81 (63–111)	84 (67–112)	69 (54.5–92)	0.075^c^
Postoperative period				
Ventilation time *median (IQR) (hours)*	11 (6–24)	19 (6–32)	10 (6–16)	0.172^c^
Length of PICU stay *median (IQR) (days)*	3 (2–5)	3 (2–6)	3 (2–4)	0.383^c^
Outcome *(Death)*	0	0	0	

a Chi-square test.

b Fisher test.

c Mann-Whitney test. TAPVR, total anomalous pulmonary venous return; PICU, pediatric intensive care unit; RACHS-1, risk adjustment for congenital heart surgery; IQR, interquartile range; CPB, cardiopulmonary bypass.

### Course of PCT levels, CRP levels, and WBC counts in the infected group and the uninfected group

**Figure 1:**
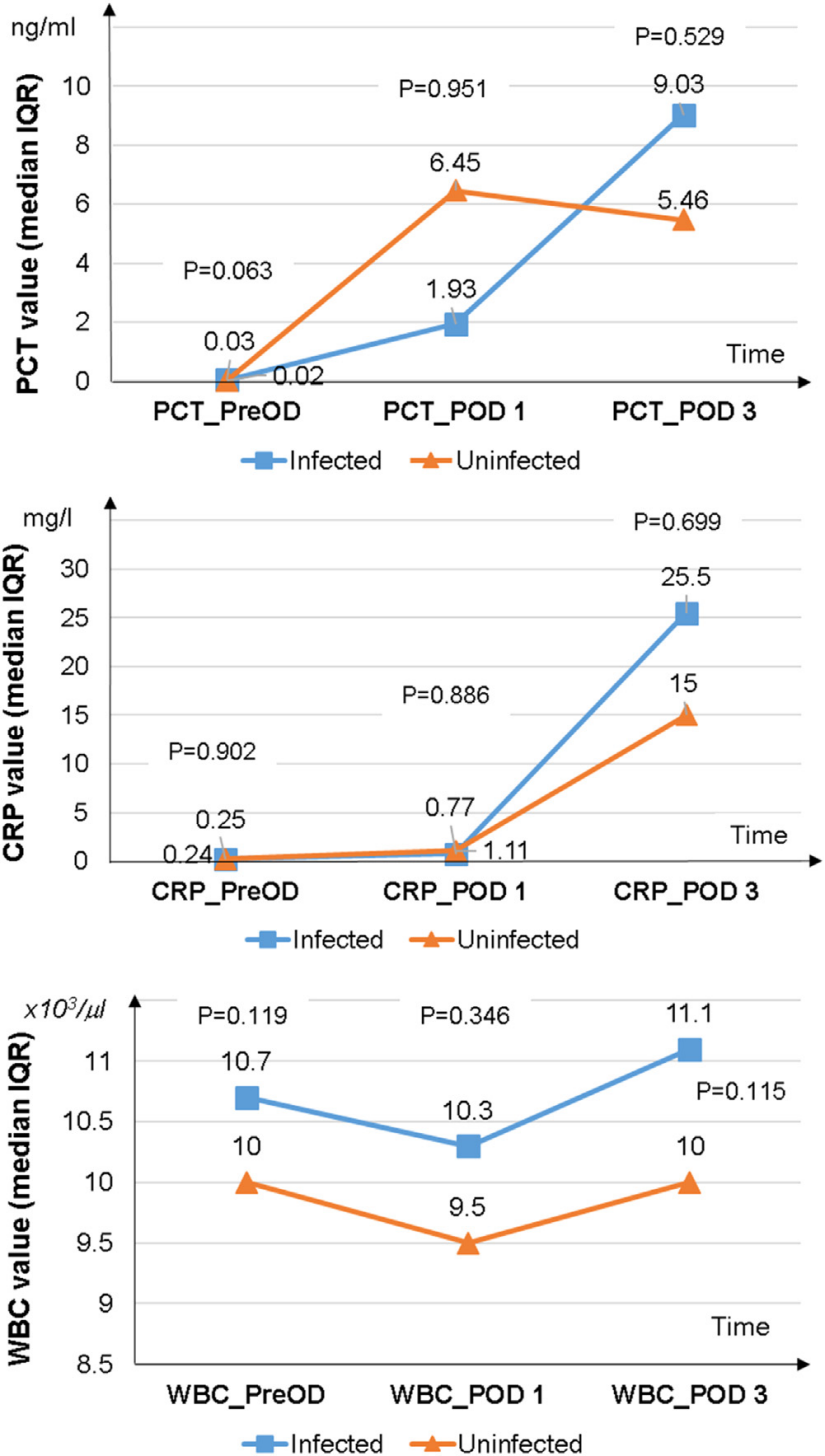
Charts comparing median PCT levels, CRP levels, and WBC counts on the preoperative day (PreOD), postoperative day 1 (POD 1), and postoperative day 3 (POD 3) between the infected and uninfected group. P-value determined with Mann-Whitney test. PCT, procalcitonin; CRP, C-reactive protein; WBC, white blood cell; IQR, interquartile range.

The median PCT levels of participants without postoperative infection increased on POD 1 but subsequently decreased by POD 3. In contrast, the median PCT levels of participants with postoperative infection showed a slight increase on POD 1, followed by a considerable rise in POD 3, reaching higher levels than those of patients without infection (9.03 ng/ml vs. 5.46 ng/ml). However, this difference was not statistically significant (*p* = 0.529) (Figure 1). Unlike the distinct trend observed in median PCT levels between the two groups, the trajectories of median CRP levels and mean WBC counts were similar between patients with and without postoperative infection. The CRP levels and WBC counts on POD 3 in the infected group were higher than those in the uninfected group (25.5 mg/l vs. 15.0 mg/l for CRP; 11.1 × 10^3^/μl vs. 10.0 × 10^3^/μl for WBC), but these differences were not statistically significant (*p* > 0.05) (Figure 1).

### Validity of models in predicting postoperative infection

Table 2 compares the validity of different models. For model 1, PCT, CRP, and WBC alone did not have significant predictive value for postoperative infection (*p* > 0.05). The cut-off points for PCT levels, CRP levels, and WBC counts on POD 3 were 4.15 ng/ml, 22.03 mg/l, and 15.3 × 10^3^/μl, respectively. For model 2, when PCT was combined with either CRP or WBC, the model showed statistically significant predictive value for postoperative infection with an AUC of 0.72 (95 % CI: 0.55–0.86) (*p* = 0.014) for the PCT and WBC combination, and an AUC of 0.71 (95 % CI: 0.57–0.86) (*p* = 0.008) for the PCT and CRP combination. Model 3, combining PCT, CRP, and WBC, had the best predictive ability for postoperative infection with an AUC of 0.81 (95 % CI: 0.69–0.93) (*p* = 0.002).

**Table 2:** Comparison of AUC, sensitivity, specificity, positive predictive value, and negative predictive value among models in predicting infection.

	AUC (95 % CI)	Sensitivity	Specificity	Positive predictive value	Negative predictive value	*P*-value
Model 1^a^						
PCT	0.67 (0.51–0.82)	48.5	70.6	76.2	41.4	0.085
CRP	0.71 (0.54–0.87)	54.6	73.3	81.8	42.3	0.072
WBC	0.65 (0.49–0.82)	60.6	62.5	76.9	43.5	0.074
Model 2						
PCT + WBC	0.72 (0.55–0.86)	66.7	68.8	81.5	50.0	0.014
PCT + CRP	0.71 (0.57–0.86)	63.6	73.3	84.0	47.8	0.008
Model 3						
PCT + CRP + WBC	0.81 (0.69–0.93)	75.8	80.0	89.3	60.0	0.002

a cut-off point for PCT = 4.15 ng/ml; cut-off point for WBC = 15.3 × 10^3^/μl; cut-off point for CRP = 22.03 mg/l (on the postoperative day 3). PCT, procalcitonin; CRP, C-reactive protein; WBC, white blood cell; AUC, area under receiver operating characteristic curve; CI, confidence interval.

**Figure 2:**
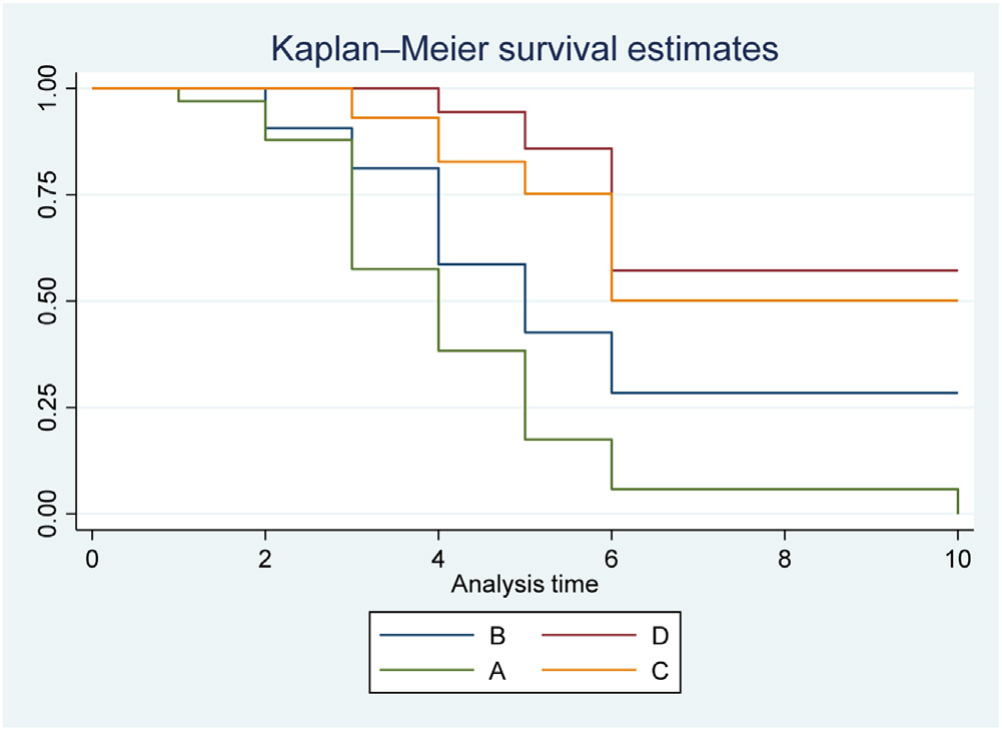
Kaplan-Meier curve analysis for predicting infection among models. A, PCT > 4.15 ng/ml (model 1). B, PCT > 4.15 ng/ml and CRP > 22.03 mg/l (model 2). C, PCT > 4.15 ng/ml and WBC > 15.3 × 10^3^/μl (model 2). D, PCT > 4.15 ng/ml, CRP > 22.03 mg/l, and WBC > 15.3 × 10^3^/μl (model 3).

Figure 2 illustrates the Kaplan-Meier survival analysis for three models to predict postoperative infection. The cut-off points for each biomarker used in the combined models were determined from the cut-off point of model 1 in the ROC curve analysis. The Kaplan-Meier plot indicates that when combining PCT level > 4.15 ng/ml and CRP level >22.03 mg/l (B) or PCT level >4.15 ng/ml and WBC count >15.3 × 10^3^/μl (C), the predictive value for postoperative infection is higher compared to PCT level >4.15 ng/ml alone (A), with a hazard ratio of 1.93 (95 % CI: 1.03–3.61) (*p* < 0.05) and 4.83 (95 % CI: 2.00–11.64) (*p* < 0.05), respectively. Model 3, with PCT level >4.15 ng/ml and CRP level > 22.03 mg/l and WBC count >15.3 × 10^3^/μl demonstrated the best predictive value for infection, with a hazard ratio 9.66 times higher (95 % CI: 2.94–31.72) than PCT >4.15 ng/ml (A) (*p* < 0.05) and 4.99 times higher (95 % CI: 1.44–17.27) than PCT > 4.15 ng/ml and CRP >22.03 mg/l (B) (*p* < 0.05).

## Discussion

Many studies have shown that PCT levels increase in patients after open-heart surgery with CPB. This increase is due to the systemic inflammatory response following CPB^15^^,^^19^^,^^26^ and the perioperative stress.^27^ The kinetics of PCT levels after open-heart surgery with CPB indicate that PCT typically rises and peaks 24 hours after surgery and returns to normal three days after surgery in uncomplicated cases.^15^^,^^20^ Continued elevation of PCT levels after POD 3 or a re-increase in POD 3 suggests postoperative infection.^14^^,^^20^^,^^26^ This study further corroborates that the trajectory of PCT levels following open-heart surgery with CPB aligns with previous findings on PCT kinetics. Procalcitonin levels in all participants after open-heart surgery with CPB were high on POD 1. However, PCT levels in uninfected patients decreased by the POD 3, while in infected patients, PCT levels continued to rise on POD 3 (Figure 1). However, to provide a more comprehensive description of PCT kinetics, it is necessary to continue monitoring PCT levels beyond the third postoperative day in future studies.

The utility of the procalcitonin test in diagnosing postoperative infection has been well-documented after open-heart surgery with CPB in many previous studies.^12^^,^^14^^,^^16^ However, recent studies have noted the uncertain role of individual PCT tests in diagnosing postoperative infection in patients undergoing open-heart surgery with CPB. Li et al., in a prospective study of 235 children undergoing open-heart surgery with CPB, found that PCT levels on POD 3 were not significantly associated with postoperative infection in multivariate analysis.^14^ Miao et al.,^18^ studying 42 children undergoing open-heart surgery with CPB, showed that the differences in PCT levels between POD 3 and POD 5 compared to POD 1 were not significantly different between the infected and uninfected groups. D'Souza et al.^17^ found no biomarker, including PCT, CRP, and WBC, within the first three postoperative days that could differentiate between infection and postoperative inflammatory response in 368 children undergoing heart surgery with CPB. Delannoy et al.^28^ showed that neither PCT nor CRP had predictive value for infection, with AUC = 0.70, *p* = 0.15, and AUC = 0.659, *p* = 0.142, respectively, in 32 adult patients undergoing open-heart surgery with CPB.

Similarly, this study did not find a statistically significant difference in the mean PCT levels on the POD 3 between the infected and uninfected groups. The predictive value of PCT alone on the POD 3 for infection showed an AUC of 0.67 (AUC of 0.67, 95 % CI: 0.51–0.82, *p* = 0.085). These findings suggest that PCT alone has limited clinical utility in predicting postoperative infection in children undergoing open-heart surgery with CPB.

There is increasing evidence that the PCT test should not be used as a stand-alone test to diagnose infection or as the gold standard for diagnosing infection.^7^^,^^8^ Although PCT is a useful biomarker, test results should be interpreted in combination with clinical assessments, other inflammatory biomarkers, and microbiological data.^9^ Studies have shown that combining PCT with other biomarkers enhances the predictive ability for infection compared to PCT alone.^22^^,^^29^ Aryafar et al.,^11^ in a study of 154 infants undergoing open-heart surgery with CPB, found that combining PCT and CRP on POD 3 had a diagnostic value for infection with an AUC of 0.90, higher than the AUC of 0.73 for PCT alone. McMaster et al.,^10^ studying 238 children undergoing open-heart surgery with CPB, reported that combining PCT and the immature-to-total neutrophil ratio improved the negative predictive value for postoperative infection compared to PCT alone. Heredia-Rodriguez et al.^23^ noted that combining PCT and WBC (four times the cut-off value) within the first three postoperative days predicted postoperative infection within 30 days of heart surgery with an AUC of 0.842 (95 % CI: 0.789–0.886) in 423 pediatric patients undergoing open-heart surgery with CPB. Rothenburger et al. showed that combining PCT levels ≥ 4 ng/ml and CRP levels ≥170 mg/l increased specificity for diagnosing infection compared to PCT alone in 563 pediatric patients after heart surgery with CPB (24). Han et al., studying 286 adult ICU patients, found that combining 24-hour PCT ≥1.5 ng/ml and 24-hour CRP ≥40 mg/l provided better diagnostic value for infection compared to PCT alone (AUC = 0.81 versus AUC = 0.78).^21^ Lamping et al., in a study of 230 adult ICU patients with SIRS/sepsis, reported that a diagnostic model combining four clinical parameters and four laboratory parameters (IL-6, platelet count, PCT, CRP) had better diagnostic value for infection than individual tests or combining CRP and PCT, with an AUC of 0.78 (95 % CI: 0.70–0.87).^22^

This study observed that in model 1, the single PCT level on POD 3 did not have statistical significance in predicting infection (Table 2). In model 2, when combining the PCT level with CRP level or WBC count on POD 3, this combination had a significant predictive value for postoperative infection. Although the AUCs of Model 2 were statistically significant, they remained below 0.8, indicating limited predictive utility. Model 3, combining the cut-off values of PCT, CRP, and WBC on POD 3, had an AUC of 0.81 (95% CI: 0.69–0.93) (*p* = 0.002). This AUC value is considered clinically useful for predicting infection. When comparing the comparative predictive abilities of the three models using Kaplan-Meier survival analysis, the study reported that the combination of PCT with CRP and WBC (PCT > 4.15 ng/ml + CRP > 22.03 mg/*l* + WBC > 15.3 × 10^3^/μl) on POD 3 offers the best predictive value for early postoperative infection compared to using PCT alone or PCT combined with CRP or WBC (Figure 2).

This study's combined biomarker model for predicting postoperative infection in open-heart surgery has an AUC value similar to those of previous studies.^11^^,^^21^^,^^22^^,^^24^ However, this model has several advantages over the models from earlier studies. First, the tests in model 3 can be commonly performed in laboratories, with quick turnaround times for results. Thus, early diagnosis of postoperative infections in patients undergoing open-heart surgery with CPB is practical and effective using model 3. Furthermore, the clearly defined biomarker thresholds enhance the model’s applicability in clinical practice. Additionally, the study notes that the PPV of model 3 is 89.3 %. This implies that among 10 cases identified as postoperative infections using this model, 9 cases would be correctly diagnosed, and 1 case might be incorrectly diagnosed. Thus, this model is useful for guiding antibiotic use in suspected postoperative infections. However, this model has a limitation: a moderate NPV of around 60 %. This implies that among 10 cases where infection is excluded by this model, 4 cases might actually be missed infections. Hence, this model may not be sufficiently robust to guide the discontinuation of antibiotic therapy.

The study also has several limitations. First, the sample size in the study is small. Biomarker monitoring was only conducted up to the third postoperative day. Adjustment for multiple comparisons was not applied. The study was conducted at a single center, and the biomarker cut-off values were derived from the ROC curve analysis of this cohort without external validation.

## Conclusions

The procalcitonin test alone is insufficient to predict infection after open-heart surgery with CPB in children. This combined biomarker model, incorporating PCT, CRP, and WBC, demonstrates promise for early postoperative infection prediction but requires further validation in larger and more diverse cohorts prior to clinical implementation.

## Authors’ contributions

Man Minh Tran, Nguyen The Nguyen Phung: Conceptualization, Methodology, Writing – review & editing. Tung Viet Le: Data curation, Formal analysis, Writing – original draft. Tuan Manh Ha: Supervision, Conceptualization, Methodology, Writing – review & editing.

## Funding

None.

## Data sharing statement

For original data, please contact the corresponding author.

## Conflicts of interest

The authors declare no conflicts of interest.

## References

[1] Tönz G.M., Kadner A., Pfammatter J.P., Agyeman P.K. (2021). Invasive bacterial and fungal infections after pediatric cardiac surgery: a single-center experience. Pediatr Infect Dis J.

[2] Yu X., Chen M., Liu X., Chen Y., Hao Z., Zhang H. (2020). Risk factors of nosocomial infection after cardiac surgery in children with congenital heart disease. BMC Infect Dis.

[3] Sochet A.A., Cartron A.M., Nyhan A., Spaeder M.C., Song X., Brown A.T. (2017). Surgical site infection after pediatric cardiothoracic surgery. World J Pediatr Congenit Heart Surg.

[4] Whiting D., Yuki K., DiNardo J.A (2015). Cardiopulmonary bypass in the pediatric population. Best Pract Res Clin Anaesthesiol.

[5] Saleem Y., Darbari A., Sharma R., Vashisth A., Gupta A. (2022). Recent advancements in pediatric cardiopulmonary bypass technology for better outcomes of pediatric cardiac surgery. Cardiothorac Surg.

[6] Goldstein B., Giroir B., Randolph A. (2005). International Consensus Conference on Pediatric Sepsis. International pediatric sepsis consensus conference: definitions for sepsis and organ dysfunction in pediatrics. Pediatr Crit Care Med..

[7] Chambliss A.B., Patel K., Colón-Franco J.M., Hayden J., Katz S.E., Minejima E. (2023). AACC guidance document on the clinical use of procalcitonin. J Appl Lab Med.

[8] Shehabi Y., Seppelt I. (2008). Pro/con debate: is procalcitonin useful for guiding antibiotic decision making in critically ill patients?. Crit Care.

[9] Wacker C., Prkno A., Brunkhorst F.M., Schlattmann P. (2013). Procalcitonin as a diagnostic marker for sepsis: a systematic review and meta-analysis. Lancet Infect Dis.

[10] McMaster P., Park D.Y., Shann F., Cochrane A., Morris K., Gray J. (2009). Procalcitonin versus C-reactive protein and immature-to-total neutrophil ratio as markers of infection after cardiopulmonary bypass in children. Pediatr Crit Care Med.

[11] Aryafar A., Di Marzio A., Guillard O., Pontailler M., Vicca S., Bojan M. (2019). Procalcitonin concentration measured within the first days of cardiac surgery is predictive of postoperative infections in neonates: a case-control study. Pediatr Cardiol.

[12] Garcia I.J., Gargallo M.B., Torné E.E., Lasaosa F.J., Viñas A.T., Tolosa C.V. (2012). Procalcitonin: a useful biomarker to discriminate infection after cardiopulmonary bypass in children. Pediatr Crit Care Med.

[13] Bavare A., Rissmiller B., Devaraj S., Guffey D., Rajapakshe D., Weiner H. (2021). Perioperative procalcitonin in predicting infection in children undergoing surgical procedures. J Surg Res.

[14] Li X., Wang X., Li S., Yan J., Li D. (2017). Diagnostic value of procalcitonin on early postoperative infection after pediatric cardiac surgery. Pediatr Crit Care Med.

[15] Farias J.S., Villarreal E.G., Dhargalkar J., Kleinhans A., Flores S., Loomba R.S. (2021). C-reactive protein and procalcitonin after congenital heart surgery utilizing cardiopulmonary bypass: when should we be worried?. J Card Surg.

[16] Fakhri D., Marwali E.M., Budiwardhana N., Roebiono P.S., Rahajoe A.U., Caesario M. (2019). Diagnosing infection after infant open heart surgery: role of procalcitonin. Asian Cardiovasc Thorac Ann.

[17] D'Souza S., Guhadasan R., Jennings R., Siner S., Paulus S., Thorburn K. (2019). Procalcitonin and other common biomarkers do not reliably identify patients at risk for bacterial infection after congenital heart surgery. Pediatr Crit Care Med.

[18] Miao Q., Chen S.N., Zhang H.J., Huang S., Zhang J.L., Cai B. (2022). A pilot assessment on the role of procalcitonin dynamic monitoring in the early diagnosis of infection post cardiac surgery. Front Cardiovasc Med.

[19] Zant R., Stocker C., Schlapbach L.J., Mayfield S., Karl T., Schibler A. (2016). Procalcitonin in the early course post pediatric cardiac surgery. Pediatr Crit Care Med.

[20] Michalik D.E., Duncan B.W., Mee R.B., Worley S., Goldfarb J., Danziger-Isakov L.A. (2006). Quantitative analysis of procalcitonin after pediatric cardiothoracic surgery. Cardiol Young.

[21] Han J.H., Nachamkin I., Coffin S.E., Gerber J.S., Fuchs B., Garrigan C. (2015). Use of a combination biomarker algorithm to identify medical intensive care unit patients with suspected sepsis at very low likelihood of bacterial infection. Antimicrob Agents Chemother.

[22] Lamping F., Jack T., Rübsamen N., Sasse M., Beerbaum P., Mikolajczyk R.T. (2018). Development and validation of a diagnostic model for early differentiation of sepsis and non-infectious SIRS in critically ill children - a data-driven approach using machine-learning algorithms. BMC Pediatr.

[23] Heredia-Rodríguez M., Bustamante-Munguira J., Lorenzo M., Gómez-Sánchez E., Álvarez F.J., Fierro I. (2017). Procalcitonin and white blood cells, combined predictors of infection in cardiac surgery patients. J Surg Res.

[24] Rothenburger M., Markewitz A., Lenz T., Kaulbach H.G., Marohl K., Kuhlmann W.D. (1999). Detection of acute phase response and infection. The role of procalcitonin and C-reactive protein. Clin Chem Lab Med.

[25] Horan T.C., Andrus M., Dudeck M.A (2008). CDC/NHSN surveillance definition of health care-associated infection and criteria for specific types of infections in the acute care setting. Am J Infect Control.

[26] Celebi S., Koner O., Menda F., Balci H., Hatemi A., Korkut K. (2006). Procalcitonin kinetics in pediatric patients with systemic inflammatory response after open heart surgery. Intensive Care Med.

[27] Minami E., Ito S., Sugiura T., Fujita Y., Sasano H., Sobue K. (2014). Markedly elevated procalcitonin in early postoperative period in pediatric open heart surgery: a prospective cohort study. J Intensive Care.

[28] Delannoy B., Guye M.L., Slaiman D.H., Lehot J.J., Cannesson M. (2009). Effect of cardiopulmonary bypass on activated partial thromboplastin time waveform analysis, serum procalcitonin and C-reactive protein concentrations. Crit Care.

[29] Lesur O., Roussy J.F., Chagnon F., Gallo-Payet N., Dumaine R., Sarret P. (2010). Proven infection-related sepsis induces a differential stress response early after ICU admission. Crit Care.

